# Assessing post‐COVID symptomatology among persons with dementia and other older adults who were hospitalized due to COVID‐19: An observational study

**DOI:** 10.1002/hsr2.1345

**Published:** 2023-07-10

**Authors:** Jennifer M. Woodward, Tsai‐Ling Liu, Marc Kowalkowski, Yhenneko J. Taylor, Bella Gutnik, Deanna A. Mangieri

**Affiliations:** ^1^ Atrium Health Senior Care, Atrium Health Charlotte North Carolina USA; ^2^ Center for Health System Sciences, Atrium Health Charlotte North Carolina USA

**Keywords:** COVID‐19, dementia, diagnostic clusters, postacute sequelae (PASC)

## 1 INTRODUCTION

The COVID‐19 pandemic has disproportionately impacted older adults with unprecedented rates of infection, hospitalization, and mortality. In the United States alone, adults aged 65 years and older account for 80% of COVID‐19 related deaths.^1^, ^2^ Public health and healthcare systems have effectively addressed acute infection demands and decreased hospitalization rates over time. However, there is growing recognition of the post‐acute sequelae of SARS‐CoV‐2 infection (PASC). Recent US‐ and UK‐based studies report 10%–25% of survivors experience lasting symptoms due to COVID‐19.^3^, ^4^ Limited data exists on the prevalence of PASC in specific subgroups such as people with dementia (PWD), who have a higher incidence of COVID‐19 and higher risk of death.^5^ This study compared PASC between PLWD and other older adults to better understand prevalent PASC symptoms and guide care in this high‐risk population.

## 2 METHODS

We used data from Atrium Health's COVID‐19 Datamart to identify 2158 patients aged 65 and older who were hospitalized for COVID‐19 between March 1, 2020, and June 30, 2021. The datamart includes data from 17 Atrium Health hospitals in North and South Carolina. Among eligible patients with multiple hospital admissions during the study period, we included only the first hospitalization for COVID‐19 (defined by a positive COVID‐19 test of nasopharyngeal swab via polymerase chain reaction [PCR] testing) to define the index study date. Patients were excluded if they tested negative on laboratory‐based tests for COVID‐19 (*n* = 705), had invalid address (due to the inability to link to area‐level socioeconomic characteristics; *n* = 136), or did not have any diagnosis within 90 days after discharge (*n* = 300) (Supporting Information: Figure S1). Health outcomes were extracted from a centralized enterprise data warehouse of medical records across the continuum of care, including outpatient practices, urgent care locations, emergency departments, and hospitals. PWD were identified using billed diagnosis codes (Supporting Information: Table S1). The primary outcome was billed diagnoses within 90 days after discharge from hospitalization. Diagnoses codes utilized at postinitial COVID‐19 diagnosis follow up appointments (*n* = 37,832) were reviewed and then grouped into common organ system clusters to better characterize post COVID‐19 symptoms. Clusters included cardiovascular, lymphatic/immune, pulmonary, musculoskeletal, renal, gastrointestinal (GI)/digestive, endocrine, neurologic, pharmacologic, hematologic, psychiatric, otolaryngological, genitourinary (GU)/reproductive, surgical, dermatologic, and other.

We compared demographic and clinical characteristics between the patient groups (patients with dementia vs. patients without dementia) using the *χ* ^2^ test or Fisher's exact test for categorical variables and Student's *t* test for continuous variables. Logistic regression was used to compare the distribution of symptom clusters between PWD and other older adult groups, adjusted for age, gender, race/ethnicity, intensive care unit (ICU) admission during hospitalization, weighted Elixhauser comorbidity index, and Multidimensional Deprivation Index. The null hypothesis (H_0_) was defined as no statistical difference in the individual risk‐adjusted outcomes of interest (i.e., each common organ system cluster) by dementia status (i.e., PWD and other older adult groups); while the alternative hypothesis (H_a_) indicates an association between dementia status and each outcome. All tests were two‐sided, and *p* < 0.05 were considered statistically significant. Analyses were conducted using SAS Version 9.4 (SAS Institute). The Atrium Health Institutional Review Board approved this study.

## 3 RESULTS

We analyzed data from 987 patients (157 PWD and 830 patients without dementia). Median age was 74. Most patients were non‐Hispanic white (67.7%), and female (56.0%). Compared to patients without dementia, PWD were older (median age 81 vs. 73) and less likely to be married (36.9% vs. 53.1%). Older adults without dementia were discharged to home more often than PWD (52.9% vs. 12.7%). In contrast, PWD had more discharges to skilled nursing facilities or home care compared to other older adults without a documented dementia diagnosis (61.1% vs 36.4%). Although ICU admissions were higher in patients without dementia compared to PWD (8.0% vs 2.5%), median length of stay (5.8 days vs. 5.0 days) and in‐hospital mortality (5.1% vs. 2.7%) were both greater for PWD (Table 1).

**Table 1:** Demographics of hospitalized COVID‐19 patients between March 1, 2020, and June 30, 2021.

	All patients	Dementia		*p* Value
		No	Yes	
All, *n* (%)	987	830 (84.1)	157 (15.9)	
Age, median (IQR)	74 (70, 80)	73 (69, 78)	81 (75, 85)	<0.001
Race/ethnicity, *n* (%)				0.130
Non‐Hispanic White	668 (67.7)	565 (68.1)	103 (65.6)	
Non‐Hispanic Black	254 (25.7)	205 (24.7)	49 (31.2)	
Hispanic	34 (3.4)	31 (3.7)	3 (1.9)	
Other	31 (3.1)	29 (3.5)	2 (1.3)	
Male, *n* (%)	434 (44.0)	379 (45.7)	55 (35.0)	0.014
Married, *n* (%)	499 (50.6)	441 (53.1)	58 (36.9)	<0.001
English speaking, *n* (%)	936 (94.8)	785 (94.6)	151 (96.2)	0.555
Medicare, *n* (%)	894 (90.6)	755 (91.0)	139 (88.5)	0.371
Discharge disposition, *n* (%)				<0.001
Home	459 (46.5)	439 (52.9)	20 (12.7)	
Home care	301 (30.5)	238 (28.7)	63 (40.1)	
Skilled nursing facility	97 (9.8)	64 (7.7)	33 (21.0)	
In‐hospital death	30 (3.0)	22 (2.7)	8 (5.1)	
Other	100 (10.1)	67 (8.1)	33 (21.0)	
ICU admission during hospitalization, *n* (%)	70 (7.1)	66 (8.0)	4 (2.5)	0.016
Length of stay (days), median (IQR)	5.1 (2.9, 10.3)	5 (2.8, 9.7)	5.8 (3.8, 15.6)	0.004
Weighted Elixhauser Comorbidity Index, median (IQR)	12 (5, 20)	11 (5, 19)	16 (10, 23)	<0.001
Multidimensional Deprivation Index, median (IQR)	55 (33, 74)	55 (35, 73)	55 (30, 76)	0.879
Minutes travel to the closest facility, median (IQR)	12.8 (9, 17.9)	13.1 (9.1, 18.3)	11.6 (8.1, 16.9)	0.074
Prior Healthcare Utilization, median (IQR)				
PCP visits	3 (2, 5)	3 (2, 5)	3 (1, 6)	0.889
Inpatient visits	0 (0, 1)	0 (0, 1)	1 (0, 2)	<0.001
ED visits	0 (0, 1)	0 (0, 1)	0 (0, 1)	0.160

Abbreviations: ED, emergency department; ICU, intensive care unit; IQR, interquartile range; PCP, primary care provider.

**Figure 1:**
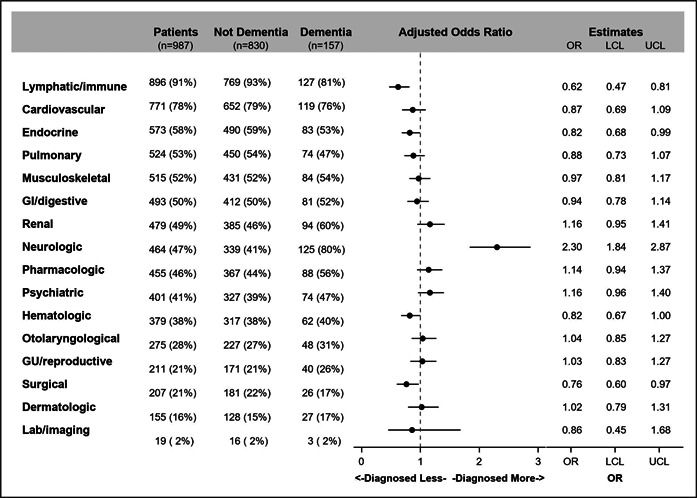
Distribution and association between dementia and each diagnostic categories within 90 days after discharge at follow‐up visits among hospitalized patients with COVID‐19. Adjusted effect estimates are depicted. Each model adjusted for age, gender, race/ethnicity, ICU admission during hospitalization, weighted Elixhauser comorbidity index, and Multidimensional Deprivation Index. The (black color) circle represents the point estimate obtained from the multivariable regression models, signifying the estimated association between dementia status and the individual outcomes of interest. The (black color) horizontal line indicates the upper and lower bounds of the 95% confidence interval around the point estimate. The (gray color) dashed vertical line indicates the line of null effect. GI, gastrointestinal; GU, genitourinary; ICU, intensive care unit; LCL, lower confidence interval; OR, odds ratio; UCL, upper confidence interval.

Most patients had lymphatic/immune diagnoses (90.8%) within 90 days after discharge, followed by cardiovascular (78.1%) and endocrine (58.1%) diagnoses. However, amongst PWD, neurologic diagnoses were the second highest (79.6%) (Figure 1). In the adjusted model, PWD were 135% more likely to have neurologic diagnoses than patients without dementia (OR = 2.35, 95% CI:1.88–2.93, *p* < 0.001). In contrast, PWD were less likely to have lymphatic/immune (OR = 0.61, 95% CI: 0.46–0.79, *p* < 0.001) diagnoses, compared to patients without dementia.

## 4 DISCUSSION

In our sample of older adults both with and without dementia, lymphatic/immune symptomatology was the most‐prominent post‐COVID hospitalization sequelae. This differs from recent findings from Hastie et al who identified cardiorespiratory symptoms, followed by confusion, as the commonest complication between 6 and 18 months in the posthospital setting.^6^ We then examined differences in post‐COVID hospitalization presentation between the general older adult population and PWD. In PWD, it is notable that the second most common cluster of symptoms was neurological changes. This parallels another study which found that mental status changes are often the presenting symptom of COVID in PWD.^7^ It also highlights the need to monitor for neurologic sequalae at subsequent follow‐up visits in PWD who had COVID‐19.

A few limitations should be noted. First, we evaluated organ system categories and clusters, grouped by diagnosis codes. While this provided a general understanding of affected organ systems, we were unable to investigate chief complaint or presenting symptoms. Moreover, there are multiple accepted strategies to group diagnosis codes by organ system or categories. Applying an alternative grouping strategy may affect the results and reproducibility of our findings. Second, we used billed diagnosis codes to identify patients with dementia, which may not capture all prevalent cases of dementia and may affect our findings and interpretations. With the estimating rates of undetected dementia between 60% and 70%,^8^, ^9^ our findings could still be helpful for the confirmed PWD. Third, although our study cohort represents a diverse population of older hospitalized adults, there are established regional differences in COVID‐19 outcomes and our findings may not generalize to older adults with COVID‐19 who were hospitalized in other geographic settings. Despite these limitations, our study is one of the few to attempt to characterize PASC in PWD. Older adults are at elevated risk of severe COVID‐19, and in PWD COVID‐19 often presents atypically with mental status changes. Our findings suggest that neurologic symptoms are a distinct post‐COVID concern among PWD compared to other older adults. Future research should further evaluate PASC symptoms to provide more insight into management. Outpatient providers can use these findings to proactively screen for neurologic sequala of COVID‐19 at follow‐up visits.

## AUTHOR CONTRIBUTIONS

**Jennifer M. Woodward**: Conceptualization; methodology; writing—original draft; writing—review & editing. **Tsai‐Ling Liu**: Conceptualization; formal analysis; funding acquisition; methodology; project administration; resources; validation; visualization; writing—original draft; writing—review & editing. **Marc Kowalkowski**: Conceptualization; methodology; supervision; visualization; writing—review & editing. **Yhenneko J. Taylor**: Conceptualization; resources; supervision; writing—review & editing. **Bella Gutnik**: Data curation; writing—review & editing. **Deanna A. Mangieri**: Conceptualization; supervision; writing—review & editing.

## CONFLICT OF INTEREST STATEMENT

The authors declare no conflict of interest.

## TRANSPARENCY STATEMENT

The lead author Jennifer M. Woodward affirms that this manuscript is an honest, accurate, and transparent account of the study being reported; that no important aspects of the study have been omitted; and that any discrepancies from the study as planned (and, if relevant, registered) have been explained.

## Supporting information

Supporting information.

Supporting information.

## Data Availability

In accordance with institutional policies and the HIPAA Privacy Rule on protected health information, data is not publicly available. The study protocol, statistical code, and fully deidentified data sets generated and/or analyzed during the current study may be available from the corresponding author on reasonable request. Dr. Tsai‐Ling Liu had full access to all of the data in this study and takes complete responsibility for the integrity of the data and the accuracy of the data analysis.
