# Supplementary figures and images for: Assessing post‐COVID symptomatology among persons with dementia and other older adults who were hospitalized due to COVID‐19: An observational study
Source: Health Sci Rep. 2023 Jul 10;6(7):e1345. doi: 10.1002/hsr2.1345 (PMC10331926; doi:10.1002/hsr2.1345)

Supplementary Figure S1. Patient selection flowchart

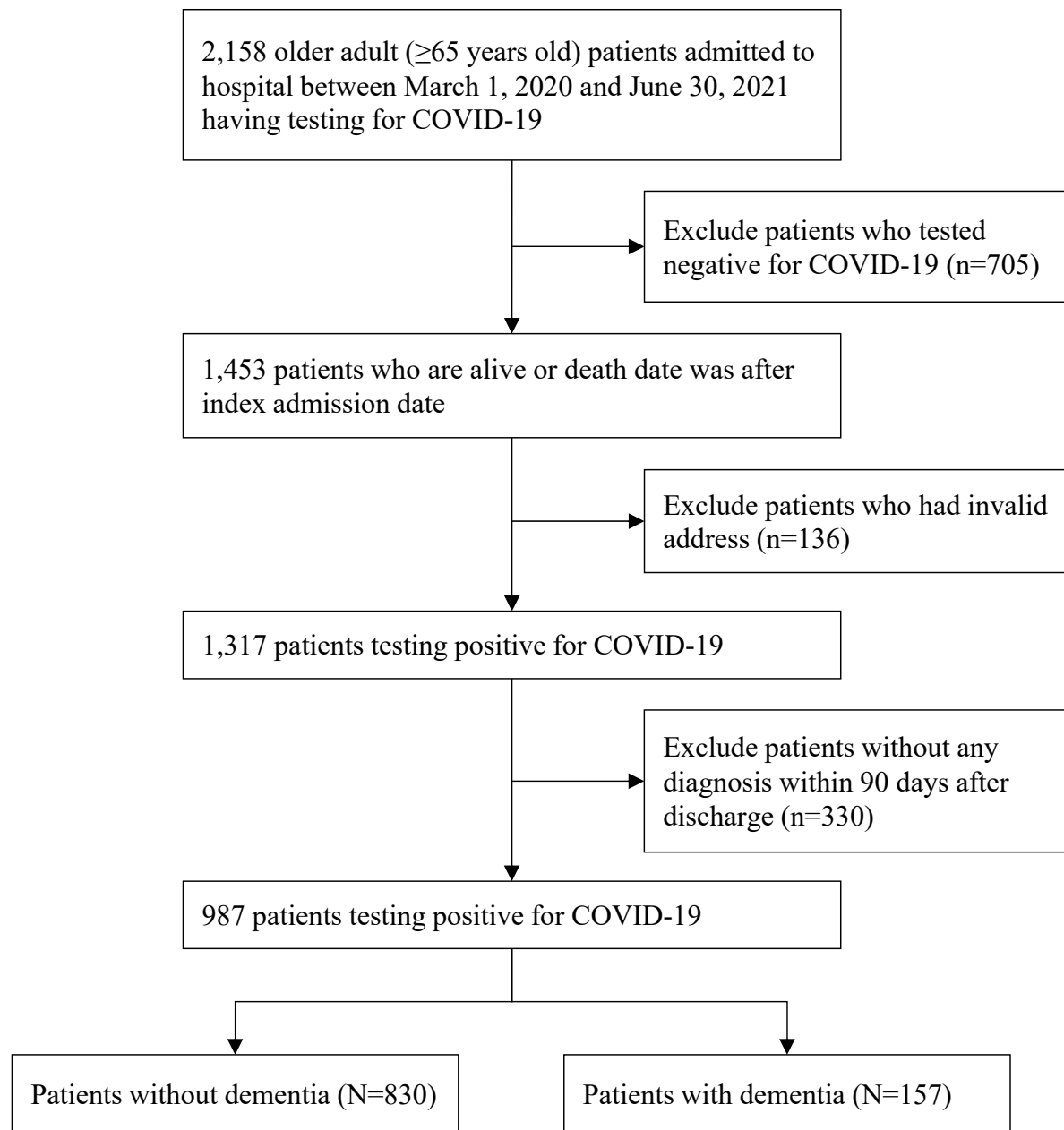

Supplement: Supplementary file 1 — Supporting information. [file HSR2-6-e1345-s001.pdf]
