# Supplementary material for: Assessing post‐COVID symptomatology among persons with dementia and other older adults who were hospitalized due to COVID‐19: An observational study
Source: Health Sci Rep. 2023 Jul 10;6(7):e1345. doi: 10.1002/hsr2.1345 (PMC10331926; doi:10.1002/hsr2.1345)
Supplement: Supplementary file 2 — Supporting information. [file HSR2-6-e1345-s002.docx]

Supplementary Table S1. List of common organ system clusters and the ICD-10 diagnoses

| Clusters | ICD-10 diagnosis codes |
| --- | --- |
| Cardiovascular | E78.00, E78.1, E78.2, E78.4, E78.49, E78.5, E78.9, I05.0, I05.2, I05.9, I07.1, I08.0, I08.1, I08.2, I08.3, I08.8, I10, I11.0, I11.9, I12.0, I12.9, I13.0, I13.10, I13.2, I16.0, I16.9, I20.0, I20.8, I20.9, I21.09, I21.19, I21.3, I21.4, I21.9, I21.A1, I24.8, I25.10, I25.110, I25.118, I25.119, I25.2, I25.41, I25.5, I25.810, I25.811, I25.82, I25.84, I25.9, I34.0, I34.1, I34.2, I34.8, I35.0, I35.1, I35.2, I35.8, I35.9, I36.1, I37.1, I42.0, I42.1, I42.2, I42.5, I42.6, I42.8, I42.9, I43, I44.0, I44.1, I44.2, I44.30, I44.4, I44.5, I44.7, I45.10, I45.2, I45.4, I45.5, I45.81, I45.89, I45.9, I46.9, I47.1, I47.2, I48.0, I48.11, I48.19, I48.2, I48.20, I48.21, I48.3, I48.4, I48.91, I48.92, I49.01, I49.1, I49.3, I49.49, I49.5, I49.8, I49.9, I50.1, I50.20, I50.21, I50.22, I50.23, I50.30, I50.31, I50.32, I50.33, I50.40, I50.41, I50.42, I50.43, I50.810, I50.811, I50.814, I50.82, I50.83, I50.84, I50.9, I51.7, I51.9, I65.22, I65.23, I65.29, I67.2, I70.0, I70.1, I70.209, I70.213, I70.90, I71.00, I71.2, I71.4, I72.9, I73.9, I74.09, I74.10, I74.5, I77.1, I77.9, I80.10, I80.3, I87.2, I87.8, I95.1, I95.3, I95.89, I95.9, Q21.1, Q25.46, Q43.8, R00.0, R00.1, R00.2, R00.8, R01.1, R03.0, R03.1, T82.838A, Z86.72, Z86.79, Z87.74 |
| Dermatology | B35.4, B35.6, B37.9, L01.00, L03.91, L05.91, L20.9, L21.9, L22, L25.8, L25.9, L27.0, L28.0, L28.2, L29.2, L29.9, L30.4, L30.8, L30.9, L40.50, L40.9, L43.8, L50.9, L53.9, L57.0, L57.8, L60.2, L60.3, L65.0, L65.9, L70.8, L71.8, L72.0, L72.3, L76.22, L81.2, L81.4, L81.9, L82.1, L84, L85.3, L89.150, L89.151, L89.152, L89.153, L89.154, L89.159, L89.214, L89.219, L89.301, L89.302, L89.309, L89.312, L89.313, L89.321, L89.322, L89.323, L89.522, L89.601, L89.613, L89.623, L89.626, L89.816, L89.819, L89.90, L89.92, L89.94, L90.5, L94.0, L97.222, L97.322, L97.409, L97.412, L97.429, L97.509, L97.512, L97.513, L97.519, L97.522, L97.524, L97.529, L97.822, L97.909, L98.429, L98.492, L98.8, L98.9, Q80.9, R20.0, R20.2, R20.8, R20.9, R21, R22.0, R22.1, R22.2, R22.31, R22.32, R22.40, R22.41, R22.42, R22.9, R23.2, R23.3, R23.8, R23.9, R61, S00.81XA, S00.83XA, S01.00XA, S01.00XD, S01.512A, S31.000A, S31.010A, S31.109A, S31.41XA, S31.831A, S40.811A, S41.112A, S41.119A, S50.811A, S51.001S, S51.009A, S51.012A, S60.511A, S61.401D, S61.419A, S70.211A, S80.211A, S80.219A, S80.811A, S80.812A, S81.012S, S81.801A, S81.812A, S81.812S, S81.819A, S91.011A, S91.109A, S91.201A, S91.209A, S91.301A, S91.302A, S91.312A, S91.312S, S91.319A, T20.00XA, T20.04XA, T24.011A, T24.012A, T24.119A, T24.211A, T24.212A, T24.219A, T30.0, T31.0, T81.30XA, T81.31XA, T81.31XD, Z87.2 |
| Endocrinology | E03.4, E03.8, E03.9, E04.1, E04.2, E04.9, E05.00, E05.20, E05.90, E06.3, E07.0, E07.89, E07.9, E08.21, E10.22, E10.65, E10.9, E11.10, E11.21, E11.22, E11.29, E11.319, E11.3299, E11.3513, E11.36, E11.39, E11.40, E11.41, E11.42, E11.43, E11.49, E11.51, E11.52, E11.59, E11.610, E11.621, E11.622, E11.649, E11.65, E11.69, E11.8, E11.9, E13.42, E13.9, E16.2, E21.0, E21.2, E21.3, E22.1, E22.2, E22.9, E27.3, E27.40, E27.8, E27.9, E28.39, E29.1, E34.9, E72.20, E74.39, E77.8, E78.6, E78.89, E79.0, E80.6, E83.19, E85.4, E88.09, E89.0, E89.2, M80.00XA, M81.0, M81.8, M84.321A, M85.80, M85.852, M85.88, M85.9, R73.01, R73.02, R73.03, R73.09, R73.9, Z13.1, Z13.820, Z86.39, Z90.411 |
| GI/digestive | D51.0, D51.9, D52.9, D53.9, E43, E44.0, E44.1, E46, E51.9, E53.1, E53.8, E55.9, E56.0, E56.9, E61.1, E63.9, E65, E66.01, E66.09, E66.2, E66.9, E67.3, E88.81, I85.00, I85.10, I86.4, I86.8, K04.7, K20.90, K21.0, K21.00, K21.9, K22.0, K22.10, K22.2, K22.4, K22.70, K22.8, K22.9, K25.0, K25.3, K25.9, K26.4, K26.9, K27.4, K27.9, K29.00, K29.41, K29.50, K29.70, K29.71, K29.81, K31.1, K31.811, K31.819, K31.84, K31.89, K31.9, K40.20, K40.90, K46.0, K46.9, K50.00, K50.012, K50.013, K50.10, K50.90, K50.913, K51.90, K52.1, K52.839, K52.9, K55.059, K55.20, K55.21, K55.9, K56.3, K56.41, K57.12, K57.30, K57.31, K57.32, K57.80, K57.90, K57.92, K58.0, K58.1, K58.9, K59.00, K59.03, K59.04, K59.09, K62.5, K62.89, K63.1, K63.2, K63.5, K64.1, K64.4, K64.8, K64.9, K65.4, K66.0, K72.00, K72.90, K72.91, K74.00, K74.4, K74.60, K74.69, K75.4, K75.81, K75.9, K76.0, K76.1, K76.3, K76.6, K76.89, K76.9, K80.10, K80.12, K80.40, K80.50, K80.70, K81.0, K81.1, K81.2, K82.0, K82.3, K82.8, K83.09, K83.8, K85.80, K85.90, K86.2, K86.89, K90.0, K92.0, K92.1, K92.2, K92.9, K94.23, Q44.6, R11.0, R11.10, R11.14, R11.15, R11.2, R13.10, R13.12, R13.13, R13.19, R14.0, R14.2, R14.3, R15.9, R16.0, R16.1, R17, R18.8, R19.00, R19.4, R19.5, R19.7, R19.8, R47.02, Z68.1, Z68.20, Z68.21, Z68.22, Z68.23, Z68.24, Z68.26, Z68.27, Z68.28, Z68.29, Z68.30, Z68.31, Z68.32, Z68.33, Z68.34, Z68.35, Z68.36, Z68.37, Z68.38, Z68.39, Z68.41, Z68.42, Z68.43, Z68.44, Z68.45, Z86.010, Z86.018, Z87.11, Z87.19 |
| GU/reproductive | K65.1, N02.9, N30.20, N30.80, N32.89, N35.919, N36.8, N39.41, N39.44, N39.46, N39.490, N39.498, N40.0, N40.1, N41.9, N45.1, N47.1, N48.83, N48.89, N50.812, N50.89, N52.9, N60.91, N62, N63.0, N63.25, N64.4, N64.9, N76.0, N81.10, N81.4, N83.202, N83.209, N83.8, N89.8, N93.9, N94.89, N94.9, N95.0, N95.2, Q55.64, R30.0, R31.0, R31.29, R31.9, R32, R33.8, R33.9, R35.0, R35.1, R35.8, R39.11, R39.12, R39.14, R39.198, R39.89, R39.9, R82.81, R84.8, R92.0, T83.021A, T83.028A, T83.9XXA, Y84.6, Z01.419, Z78.0, Z87.448, Z90.12, Z90.710, Z90.711, Z90.722 |
| HEENT | G36.0, H00.016, H04.123, H04.129, H05.239, H10.10, H10.13, H10.32, H10.402, H10.9, H11.153, H11.31, H11.32, H20.9, H25.811, H25.812, H26.9, H35.00, H35.30, H40.2294, H40.9, H49.20, H49.22, H49.882, H50.10, H50.9, H53.469, H53.8, H53.9, H54.3, H54.40, H54.62, H54.7, H54.8, H57.10, H57.89, H60.90, H60.93, H61.20, H61.21, H61.22, H61.23, H66.92, H69.80, H81.10, H81.399, H83.09, H83.2X9, H90.0, H90.3, H90.5, H91.13, H91.90, H91.91, H91.92, H92.02, H93.11, H93.19, H93.3X9, H93.8X9, I69.322, I69.328, I69.391, J30.2, J30.89, J30.9, J31.0, J32.9, J33.0, J33.9, J34.89, J34.9, J38.00, J38.3, K02.9, K05.10, K05.30, K05.6, K08.109, K08.3, K08.89, K08.9, K11.20, K11.21, K12.0, K12.1, K12.30, K13.0, K13.70, K13.79, K14.0, R04.0, R04.2, R05, R09.81, R13.11, R40.0, R42, R43.8, R47.01, R47.81, R47.82, R47.89, R47.9, R63.3, S00.31XA, S00.531A, S02.2XXA, S02.30XA, S02.31XA, S02.32XA, S02.5XXA, S05.01XA, S09.92XA, T16.9XXA, Z01.00, Z98.41, Z98.42 |
| Hematology | C10.9, C16.9, C18.7, C18.9, C21.0, C22.0, C22.1, C23, C26.9, C32.1, C34.11, C34.2, C34.31, C34.32, C34.90, C34.91, C34.92, C43.59, C43.70, C43.71, C44.320, C44.40, C44.42, C44.519, C44.90, C44.91, C44.92, C49.A0, C49.A2, C50.211, C50.212, C50.312, C50.411, C50.412, C50.511, C50.911, C50.912, C50.919, C54.1, C55, C61, C64.1, C64.9, C67.8, C67.9, C73, C77.1, C78.00, C78.01, C78.02, C78.5, C78.6, C78.7, C78.89, C79.11, C79.31, C79.51, C79.62, C79.9, C7A.011, C80.1, C81.90, C82.90, C83.00, C83.10, C83.30, C83.39, C83.50, C85.90, C86.5, C88.0, C88.4, C90.00, C91.10, C91.11, C91.60, C91.90, C92.00, C92.02, C92.10, C92.90, C93.10, C95.90, D04.39, D04.5, D05.10, D09.0, D12.2, D12.3, D12.4, D12.5, D12.6, D12.7, D12.8, D15.1, D16.9, D17.0, D17.39, D18.00, D22.9, D24.1, D27.1, D32.0, D35.2, D3A.00, D3A.019, D3A.020, D3A.8, D45, D46.1, D46.20, D46.9, D46.Z, D47.2, D47.3, D48.5, D48.9, D49.0, D49.2, D50.0, D50.9, D56.9, D59.10, D59.12, D61.810, D61.811, D61.818, D62, D63.0, D63.1, D63.8, D64.89, D64.9, D65, D66, D68.0, D68.2, D68.32, D68.51, D68.59, D68.61, D68.9, D69.2, D69.3, D69.49, D69.59, D69.6, D70.1, D70.3, D70.8, D70.9, D72.10, D72.19, D72.810, D72.818, D72.819, D72.820, D72.829, D72.9, D73.5, D75.1, D75.82, D75.89, D76.1, D80.1, E83.110, E83.119, E88.3, G89.3, J91.0, Z08, Z12.4, Z12.83, Z85.038, Z85.048, Z85.060, Z85.110, Z85.118, Z85.21, Z85.3, Z85.41, Z85.42, Z85.46, Z85.51, Z85.528, Z85.6, Z85.72, Z85.79, Z85.818, Z85.820, Z85.828, Z85.831, Z85.840, Z85.848, Z85.850, Z85.89, Z85.9 |
| Lab/imaging | R39.15, R71.0, R71.8, R79.82, R82.998, R90.89, R93.421, R94.2 |
| Lymphatic/immune | A04.71, A04.72, A08.39, A09, A31.0, A41.01, A41.4, A41.51, A41.52, A41.59, A41.81, A41.89, A41.9, A49.02, A49.1, A49.8, B00.1, B00.9, B02.23, B02.9, B17.9, B18.2, B19.10, B20, B25.9, B33.22, B34.2, B34.9, B35.1, B35.3, B36.9, B37.0, B37.2, B37.3, B37.81, B44.0, B44.1, B44.7, B44.9, B86, B94.8, B95.2, B95.61, B95.62, B95.7, B95.8, B96.1, B96.20, B96.3, B96.4, B96.5, B97.21, B97.29, B97.81, B97.89, D84.821, D84.9, D89.89, D89.9, G93.3, I00, I01.1, I30.8, I31.3, I33.0, I38, I51.4, I96, J00, J01.00, J01.01, J01.90, J02.8, J02.9, J03.90, J04.0, J06.9, J12.81, J12.82, J12.89, J12.9, J15.0, J15.1, J15.211, J15.6, J15.8, J15.9, J16.8, J18.1, J18.9, J20.8, J20.9, J32.0, J40, J47.0, J47.9, J61, J69.0, K12.2, K55.30, L02.211, L02.214, L02.31, L02.32, L02.419, L02.92, L03.032, L03.113, L03.114, L03.115, L03.116, L03.119, L03.211, L03.314, L03.317, L03.90, L08.9, M00.861, M00.9, M46.1, M46.28, M86.18, M86.8X3, M86.8X7, M86.9, N30.00, N30.81, N30.90, N39.0, N41.0, R50.81, R50.9, R65.10, R78.81, R82.71, T78.40XA, T81.41XA, T81.43XA, T81.49XA, T82.6XXA, T83.518A, T83.61XA, T84.59XA, T84.7XXA, T87.44, U07.1, Y95, Z20.822, Z20.828, Z21, Z22.322, Z77.120, Z86.14, Z86.16, Z86.19, Z86.2, Z87.01, Z87.440, Z87.442, Z87.892, Z88.0, Z88.1, Z88.2, Z88.5, Z88.6, Z88.8, Z91.010, Z91.011, Z91.012, Z91.013, Z91.018, Z91.030, Z91.040, Z91.041, Z91.048, Z91.09, Z91.128, Z92.25, Z94.81 |
| Musculoskeletal | D86.0, D86.3, D86.9, E85.89, E85.9, G56.00, G56.01, G56.02, G56.03, G56.20, G56.23, G57.90, G89.11, G89.18, G89.29, G89.4, I73.00, M02.30, M05.79, M05.80, M05.9, M06.00, M06.042, M06.09, M06.4, M06.9, M10.09, M10.9, M11.232, M11.261, M12.812, M13.879, M15.0, M15.4, M15.8, M15.9, M16.0, M16.10, M16.11, M16.12, M16.9, M17.0, M17.10, M17.11, M17.12, M17.4, M17.9, M18.12, M19.011, M19.012, M19.019, M19.021, M19.029, M19.031, M19.032, M19.041, M19.042, M19.049, M19.071, M19.072, M19.90, M1A.3410, M1A.4720, M1A.9XX0, M1A.9XX1, M21.332, M21.371, M21.372, M21.962, M25.312, M25.461, M25.471, M25.473, M25.50, M25.511, M25.512, M25.519, M25.521, M25.531, M25.532, M25.539, M25.551, M25.552, M25.559, M25.561, M25.562, M25.569, M25.571, M25.572, M25.579, M25.611, M25.619, M25.711, M25.78, M26.621, M30.1, M32.9, M34.9, M35.00, M35.01, M35.2, M35.3, M35.9, M41.84, M41.9, M43.10, M43.16, M43.17, M43.26, M46.92, M46.96, M47.12, M47.22, M47.26, M47.812, M47.814, M47.815, M47.816, M47.817, M47.819, M47.896, M47.897, M47.9, M48.00, M48.02, M48.04, M48.061, M48.062, M48.07, M48.50XA, M48.54XA, M48.56XA, M48.56XD, M50.10, M50.20, M50.30, M50.321, M51.16, M51.26, M51.34, M51.35, M51.36, M51.37, M51.9, M53.3, M53.82, M54.10, M54.12, M54.16, M54.17, M54.2, M54.30, M54.32, M54.40, M54.5, M54.6, M54.81, M54.89, M54.9, M60.9, M62.08, M62.551, M62.81, M62.82, M62.838, M62.84, M62.89, M65.30, M67.20, M67.432, M70.31, M70.61, M70.62, M71.21, M71.9, M72.0, M75.00, M75.02, M75.101, M75.121, M75.122, M75.22, M75.32, M75.41, M75.42, M75.51, M75.82, M76.01, M76.02, M76.30, M76.31, M77.11, M77.40, M79.1, M79.10, M79.12, M79.18, M79.2, M79.5, M79.601, M79.602, M79.603, M79.604, M79.605, M79.606, M79.631, M79.639, M79.641, M79.642, M79.644, M79.645, M79.651, M79.661, M79.662, M79.669, M79.671, M79.672, M79.673, M79.674, M79.675, M79.676, M79.7, M85.89, M94.0, M96.1, Q76.49, R07.0, R07.2, R07.81, R07.89, R07.9, R10.10, R10.11, R10.12, R10.13, R10.2, R10.30, R10.31, R10.32, R10.33, R10.817, R10.84, R10.9, R26.0, R26.2, R26.81, R26.89, R26.9, R27.0, R27.8, R29.6, R29.898, R52, S05.10XA, S13.4XXA, S13.9XXA, S16.1XXA, S20.211A, S20.212A, S20.219A, S22.000A, S22.080A, S22.089A, S22.31XA, S22.39XA, S29.9XXA, S30.0XXA, S30.1XXA, S32.000A, S32.010A, S32.020A, S32.409A, S32.592A, S39.92XA, S40.029A, S42.201A, S42.201D, S42.211A, S42.213A, S42.291A, S42.293A, S42.354D, S42.91XA, S43.012A, S46.002A, S46.009A, S50.00XA, S50.02XA, S52.351D, S52.502A, S52.502D, S52.591A, S52.90XA, S52.91XA, S62.101A, S62.102A, S62.109A, S62.112A, S63.042A, S69.90XA, S69.91XA, S70.01XA, S70.02XA, S70.11XA, S72.001A, S72.001D, S72.002A, S72.012A, S72.141A, S72.142D, S72.92XA, S72.92XD, S76.011A, S76.311A, S80.00XA, S80.01XA, S80.02XA, S80.10XA, S80.11XA, S80.12XA, S82.831D, S82.841D, S82.842A, S82.842D, S82.852A, S82.852D, S82.891D, S82.892A, S83.91XA, S83.92XA, S86.812A, S89.90XA, S90.32XA, S92.351A, S93.401A, S93.402A, S93.402D, S93.409A, S93.609A, S98.139A, S99.921A, S99.922A, T14.8XXA, T14.90XD, T83.84XA, V03.00XA, W01.0XXA, W01.10XA, W01.190A, W01.198A, W05.0XXA, W06.XXXA, W07.XXXA, W10.8XXA, W18.11XA, W18.2XXA, W18.30XA, W18.30XD, W18.39XA, W19.XXXA, W19.XXXD, W19.XXXS, W20.8XXA, W22.09XA, W22.8XXA, Z87.39, Z87.81, Z89.021, Z89.022, Z89.421, Z89.422, Z89.431, Z89.512, Z89.612, Z91.81 |
| Neurology | F01.50, F01.51, F02.80, F02.81, F03.90, F03.91, F05, F51.01, F51.3, F51.4, F51.9, G11.8, G11.9, G20, G21.4, G24.4, G25.0, G25.3, G25.5, G25.81, G25.9, G30.1, G30.9, G31.83, G31.84, G31.89, G31.9, G35, G37.3, G40.019, G40.111, G40.209, G40.319, G40.409, G40.901, G40.909, G43.009, G43.709, G43.909, G44.219, G45.9, G46.0, G46.4, G47.00, G47.09, G47.10, G47.19, G47.20, G47.30, G47.31, G47.33, G47.34, G47.36, G47.37, G47.61, G47.8, G47.9, G50.0, G51.0, G54.1, G54.6, G56.32, G57.30, G57.31, G57.92, G57.93, G58.8, G58.9, G60.8, G60.9, G61.81, G62.9, G65.2, G70.00, G72.41, G72.81, G81.11, G81.90, G81.91, G81.94, G82.50, G83.24, G90.9, G91.2, G92, G93.0, G93.1, G93.40, G93.41, G93.49, G93.6, G93.89, G93.9, G95.29, I60.9, I61.1, I61.4, I61.9, I62.00, I62.03, I62.9, I63.239, I63.29, I63.312, I63.40, I63.412, I63.449, I63.511, I63.512, I63.81, I63.89, I63.9, I66.02, I66.23, I66.9, I67.82, I67.89, I67.9, I68.0, I69.298, I69.30, I69.311, I69.320, I69.351, I69.354, I69.359, I69.390, I69.393, I69.398, I69.90, I69.993, R06.83, R25.1, R27.9, R29.704, R29.707, R29.718, R29.721, R29.723, R29.728, R29.810, R29.818, R29.90, R40.20, R40.2142, R40.2143, R40.2243, R40.2252, R40.2362, R40.2363, R40.2412, R40.2420, R40.2432, R41.0, R41.3, R41.81, R41.82, R41.841, R41.89, R47.1, R48.2, R51, R51.9, R53.2, R90.82, S00.03XA, S00.11XA, S00.12XA, S01.90XA, S06.0X9A, S06.5X0A, S06.5X9A, S06.5X9D, S06.5X9S, S09.90XA, Z72.820, Z86.12, Z86.69, Z86.73, Z87.820 |
| Pharmacology | G24.01, G62.0, T36.0X5A, T36.8X5A, T36.95XA, T37.8X5A, T38.0X5A, T38.0X5D, T38.3X5A, T40.2X5A, T40.2X5D, T40.605A, T42.6X5A, T42.75XA, T45.1X1A, T45.1X5A, T45.1X5D, T45.2X1A, T45.511A, T45.515A, T45.516A, T45.8X5A, T46.4X5A, T50.1X5A, T50.1X6A, T50.2X5A, T50.901A, T50.905A, T50.911A, T50.Z95A, X08.8XXA, Z03.818, Z16.12, Z16.21, Z16.24, Z77.090, Z79.01, Z79.02, Z79.1, Z79.2, Z79.4, Z79.51, Z79.52, Z79.811, Z79.82, Z79.83, Z79.84, Z79.890, Z79.891, Z79.899, Z92.21, Z92.29 |
| Psychiatric | F06.31, F09, F10.10, F10.11, F10.20, F10.99, F11.20, F11.23, F11.90, F12.11, F13.20, F17.200, F17.209, F17.210, F17.211, F17.220, F17.290, F19.90, F20.9, F25.9, F31.10, F31.32, F31.61, F31.81, F31.9, F32.0, F32.1, F32.5, F32.89, F32.9, F33.0, F33.1, F33.2, F33.3, F33.40, F33.41, F33.42, F33.9, F34.1, F34.9, F39, F41.0, F41.1, F41.8, F41.9, F42.3, F42.9, F43.0, F43.10, F43.20, F43.21, F43.22, F43.23, F43.9, F45.42, F48.2, F48.8, F51.02, F51.04, F84.0, F90.9, F98.8, F99, H93.293, K70.30, R44.0, R44.1, R44.3, R45.1, R45.4, R45.5, R45.6, R45.86, R45.89, R46.89, Z71.6, Z72.0, Z77.22, Z81.8, Z86.59, Z87.891 |
| Pulmonary | I26.92, I26.93, I26.99, I27.0, I27.20, I27.21, I27.23, I27.29, I27.81, I27.82, I28.8, I74.3, I81, I82.401, I82.402, I82.403, I82.409, I82.411, I82.412, I82.419, I82.431, I82.432, I82.439, I82.441, I82.442, I82.452, I82.4Y1, I82.509, I82.542, I82.611, I82.612, I82.619, I82.621, I82.90, I82.B11, I83.90, J39.8, J42, J43.0, J43.1, J43.2, J43.8, J43.9, J44.0, J44.1, J44.9, J45.20, J45.21, J45.30, J45.40, J45.41, J45.50, J45.51, J45.901, J45.909, J45.998, J80, J82, J82.82, J82.83, J84.01, J84.10, J84.112, J84.113, J84.114, J84.89, J84.9, J85.0, J85.2, J86.9, J90, J91.8, J93.0, J93.82, J93.83, J93.9, J94.8, J95.1, J95.831, J95.851, J96.00, J96.01, J96.02, J96.10, J96.11, J96.12, J96.20, J96.21, J96.22, J96.90, J96.91, J96.92, J98.01, J98.09, J98.11, J98.2, J98.4, J98.8, J98.9, R06.00, R06.01, R06.02, R06.03, R06.09, R06.2, R06.4, R06.81, R06.82, R06.89, R09.02, R09.1, R09.2, R91.1, R91.8, T17.908A, T17.998A, T79.7XXA, Z86.711, Z86.718, Z87.09 |
| Renal | E83.39, E83.41, E83.42, E83.51, E83.52, E86.0, E86.1, E86.9, E87.0, E87.1, E87.2, E87.3, E87.4, E87.5, E87.6, E87.70, E87.71, E87.8, J81.0, J81.1, N04.1, N04.9, N05.1, N05.9, N12, N13.0, N13.1, N13.2, N13.30, N13.8, N13.9, N17.0, N17.9, N18.1, N18.2, N18.3, N18.30, N18.31, N18.32, N18.4, N18.5, N18.6, N18.9, N19, N20.0, N20.1, N20.2, N25.0, N25.81, N26.1, N27.1, N28.0, N28.1, N28.89, N28.9, N31.9, N32.81, N36.2, R34, R57.1, R57.8, R57.9, R58, R60.0, R60.1, R60.9, R65.20, R65.21, R74.0, R74.01, R74.8, R76.8, R77.8, R79.0, R79.1, R79.81, R79.89, R80.9, R81, R82.90, R94.4, R94.5, R94.6, T86.19, Z91.15 |
| Surgery | I95.81, I97.89, K35.32, K35.33, K36, K42.9, K43.0, K43.9, K44.9, K56.51, K56.600, K56.609, K56.690, K56.699, K56.7, K80.20, K80.71, K80.80, K81.9, K91.89, L76.32, M96.89, R50.82, S01.01XA, S01.81XA, T81.42XA, T81.83XA, T81.89XA, T81.89XD, T82.818A, T82.857A, T82.9XXA, T85.590A, Y79.2, Y82.8, Y83.0, Y83.2, Y83.5, Y83.8, Y83.9, Z01.810, Z01.811, Z01.812, Z01.818, Z46.82, Z90.81, Z95.810, Z95.818, Z95.828, Z96.611, Z96.612, Z96.641, Z96.642, Z96.643, Z96.649, Z96.651, Z96.652, Z96.653, Z96.659, Z98.890 |
| Other | E88.2, G96.08, G97.1, I46.2, I51.3, I51.89, I65.21, I67.1, I69.318, I69.319, I70.202, I70.25, I70.262, I70.434, I72.2, I77.0, I77.810, I77.819, I80.209, I80.239, I83.019, I83.029, I83.813, I83.93, I88.9, I89.0, I89.1, I99.8, I99.9, M79.81, M79.89, Q26.6, Q27.30, Q28.3, Q92.8, R09.89, R25.2, R29.709, R29.719, R40.2141, R40.2251, R40.2361, R53.1, R53.81, R53.82, R53.83, R54, R55, R56.9, R57.0, R59.0, R59.1, R59.9, R62.51, R62.7, R63.0, R63.4, R63.5, R63.8, R64, R68.0, R68.2, R68.83, R68.89, R69, R76.11, R89.9, R93.0, R93.1, R93.2, R93.3, R93.5, R93.8, R93.89, R94.01, R94.31, R94.39, R97.20, S35.299A, S75.009A, T67.01XA, T68.XXXA, T70.29XA, T81.11XA, T81.718A, T82.524A, T83.018A, T83.031A, T83.83XA, T87.54, T87.81, T87.89, T87.9, T88.1XXA, V87.7XXA, V89.2XXA, W54.1XXA, W55.03XA, X10.0XXA, X30.XXXA, X50.1XXA, X58.XXXA, X58.XXXD, X58.XXXS, Y65.8, Y71.8, Y84.8, Y92.002, Y92.003, Y92.009, Y92.012, Y92.122, Y92.128, Y92.129, Y92.230, Y92.234, Y92.239, Y92.524, Y93.01, Y93.89, Y93.E1, Z00.00, Z01.84, Z01.89, Z02.89, Z03.89, Z04.1, Z04.3, Z04.9, Z09, Z11.1, Z11.52, Z11.59, Z11.9, Z12.11, Z12.2, Z12.31, Z12.39, Z12.5, Z13.0, Z13.21, Z13.29, Z13.31, Z13.6, Z13.9, Z14.8, Z15.01, Z15.09, Z15.89, Z16.35, Z17.0, Z23, Z29.9, Z3A.19, Z43.0, Z43.1, Z45.010, Z45.018, Z45.2, Z46.59, Z46.6, Z47.1, Z47.81, Z47.89, Z48.02, Z48.21, Z48.815, Z48.89, Z51.0, Z51.11, Z51.12, Z51.5, Z51.81, Z51.89, Z53.20, Z53.21, Z53.29, Z53.8, Z53.9, Z57.9, Z59.0, Z59.4, Z59.9, Z60.2, Z60.9, Z63.4, Z63.6, Z65.9, Z66, Z71.0, Z71.2, Z71.3, Z71.89, Z72.4, Z72.89, Z73.3, Z74.01, Z74.09, Z74.1, Z75.1, Z76.0, Z76.89, Z78.1, Z78.9, Z80.0, Z80.1, Z80.3, Z80.42, Z80.51, Z80.8, Z80.9, Z82.0, Z82.3, Z82.49, Z82.5, Z82.61, Z82.62, Z83.0, Z83.3, Z83.71, Z86.74, Z87.828, Z87.898, Z90.10, Z90.11, Z90.13, Z90.2, Z90.49, Z90.5, Z90.79, Z91.14, Z91.19, Z91.410, Z91.89, Z92.3, Z92.82, Z92.89, Z93.0, Z93.1, Z93.2, Z93.6, Z94.0, Z94.1, Z94.84, Z95.0, Z95.1, Z95.2, Z95.3, Z95.5, Z95.820, Z96.0, Z96.1, Z96.41, Z96.82, Z96.89, Z98.1, Z98.2, Z98.49, Z98.51, Z98.61, Z98.84, Z99.11, Z99.2, Z99.3, Z99.81, Z99.89 |
